# Supplementary material for: Estimating the Spatial Accessibility to Blood Group and Rhesus Type Point-of-Care Testing for Maternal Healthcare in Ghana
Source: Diagnostics (Basel). 2019 Nov 5;9(4):175. doi: 10.3390/diagnostics9040175 (PMC6963207; doi:10.3390/diagnostics9040175)
Supplement: Supplementary file 1 [file diagnostics-09-00175-s001.pdf]

## Supplementary file 1: Procedure for estimating the travel time

To begin this, a cost surface algorithm was designed with these parameters: a grid cell of size 10m was assigned to the spatial features and values were then assigned to the predetermined grids. The essence of this was to grant the user control over travel time estimation. Every image file is divided into grids or cells and as a strategy to determine travel time, these grids or cells are leveraged on to calculate how much users should travel and the equivalent of that in time. In essence, to travel a 10m grid of roads, the travel time on roads and the medium of travel is considered and this eventually determines the value of time for the 10m grid of road. This concept applies for the other land cover features. Based on this logic and the following algorithm roads were assigned low values because traveling on roads is faster than travel via paths or impediments. Assigning of values to spatial features was done via the map algebra tool and because cost distance requires the cost surface dataset and the source, the calibrated spatial dataset served as the cost surface dataset and referral hospitals served as the source for calculating the cost distance. The output is a map showing shortest travel time (cell by cell) from any point in the map to referral hospitals in the region. It is imperative that the right cell size be determined because it impacts the results of the study. The cell size must be small enough to capture the details on the image and it should not be too large to affect the efficiency of processing the algorithm or model.

Algorithms which allowed for carrying out conversion of data from vector to raster, map algebra (cost surface models) and cost distance was developed using Python 2.7. Designing the algorithm helped to avoid developing multiple algorithms for the varying districts. Based on information gathered, the identified (most common) mode of travel was the tricycle and the travel speed was pecked at 20 km/h/. This served as a guide for determining travel time in the region.

The process of calculating travel time leveraged on the cell size of 10 meters and in order to sync the units, our determined travel time of 20km/h was converted to 20,000 meters. In order to estimate how many seconds it takes to travel 1 meter, a new unit of meters per second (m/s) had to be established. Seeing that 3,600 seconds make an hour, it was divided by 20,000 meters which equals 0.18 seconds. Our cell size was set at 10 meters so to find out how long it takes to travel each cell we multiplied 0.18 seconds by 10. This meant it took 1.8 seconds to travel via road using the tricycle. The raster calculator was then used in reassigning each cell to 1.8 seconds as the travel time required to travel each cell. This algorithm was applied to the other land cover types which includes the DEM data set, paths and rivers.

## Supplementary file 2: Distance/travel time categorisation of the towns and their names

### Less than 10 kilometers / 30 minutes

- AKOMKONGU
- ASONGE
- BALUNGU
- BANSUNDE
- BAWKU
- BINABA
- BOGOROGO
- BOLGATANGA
- BONGO
- BOYAA
- DABILA
- DAKEO
- DUA
- FEO
- GAMBIBGU
- GARU
- GUNYORO
- GYNTIGA
- KAGBIRI
- KALBEO
- KANDIGA
- KAYILO
- KOBURI
- KONGO
- KOSANABA
- KPATIA
- KUBONGO
- KUGURAGO
- KULUNGUGU
- KULWASE
- MANYORO
- MELIGA
- MIRIGU
- MISSIGA
- MOGNORI
- NABISI
- NAGA
- NAGTUNA
- NAKOLO
- NAMON
- NANGODE
- NASIA
- NAVRONGO
- NINKONGO
- PAGA
- PELUNGU
- PINDA
- PULIMAKOM
- PUNGU
- SAMBULUNGU
- SANDEMA
- SEKOTI
- SHEAGA
- SHERIGU
- SHIA
- SINENSI
- SIRIGU
- SOE
- SOKOBO
- SSNIT FLAT
- SUMBRUNGU
- TARIUAGO
- TESHIE
- TIMONDE
- TIMPANE
- TONGO
- TONGO SHRINE
- WEDENA
- WIAGA
- WIDENABA
- WINKOGO
- WORRICAMBO
- YARIGUGU
- YELWOKO
- YINDURI
- YOROGO
- YUWA
- ZAARE
- ZABUGU
- ZALERIGU
- ZARRE
- ZEBILLA
- ZESERI
- ZOKO
- ZONGOYIRI
- ZORSI
- ZUARUNGU

### Between 10 and 15 kilometers (30 and 45 minutes)

- ATOBA
- BANSI
- BAZUA
- BIDURI
- BIU
- BUGRI
- CHIANA
- CHUCHULIGA
- DATOKO
- KAADI
- KATIU
- KOIKPIRA
- KOKORE
- KOMNAB TINGA
- KOWMOKA
- KUGRI
- KUKA TAMBALIGE
- NAFKULIGA
- PUSIGA
- PWALUGU
- SIKIRI
- SOMFUSI
- SUGUDI
- TILLI
- WIRINYANGA

### Between 15 and 20 kilometers (45 and 60 minutes)

| | | | |
|---|---|---|---|
| BIUN | GWADEMA | NAKONG | SESHIE |
| DONINGA | KADEMA | SAPELIGA | SINEBAGA |

**Between 20 and 25 kilometers (60 and 75 minutes)**

BECHUASE · KANJARGA · NUNGO · UWASI

CHANSA · KAYORO · TULA

**Greater than 25 kilometers (75 minutes)**

BECHISI · FUMBISI · GWEDEMBISA · WIASI
